# Supplementary material for: How the banking system is creating a two-way inflation in an economy
Source: PLoS One. 2020 Apr 2;15(4):e0229937. doi: 10.1371/journal.pone.0229937 (PMC7117694; doi:10.1371/journal.pone.0229937)
Supplement: Attachment — Submitted filename: Response-2-Reviewers.pdf [file pone.0229937.s002.pdf]

Response to Reviewer

Reviewer 1:

1. Introduction is now segregated into three paragraphs: First one sketches the interaction between nominal deposit rate and demand pull inflation. Second paragraph briefly describes the inter-relation between nominal lending rate and cost push inflation. Finally, paragraph 3 combines the arguments presented in paragraph 1 & 2 and discusses how the article is organized.
2. Section: 2 is now thematically organized around 5 paragraphs each focusing on different clusters of empirical studies regarding Fisher effect.
3. Two more paragraphs about latest state of the art research regarding Fisher effect are appended. Studies earlier than 2002 are cited only to follow the chronological ordering of literature since the inception of Fisher effect in as early as 1930s.
4. Beta represents the portion of credit of those borrowers who do not have deposits with any bank. In practical cases, any customer who has a credit line is also supposed to have a depository account with the bank. Every borrower must have to have either a current/savings/SND account with his/her lending partner. At this point, Beta can be reasonably set to zero. However, the current/savings/SND account of the borrower may have zero balance theoretically. In this hypothetical but plausible scenario, Beta can not be zero.
5. We are concerned here with the Aggregate Demand – Aggregate Supply (AD-AS) model. Hence, Q represents total output.

5.1. EM and EC are indeed the two sides of the same coin: EM faces the depositors while EC faces the borrowers (producers). EM is actually a portion of nominal interest income while EC corresponds to a portion of nominal interest expense. Like every other transaction, payment of interest involves two parties: One pays and another receives. Hence, changing EM will change the depositors' income and thus affects aggregate demand curve accordingly. On the other hand, changes in EC will be translated into a change in cost of production (as interest expense is usually considered as a cost of production. See for example, [1], [2], [3]). As cost of production changes so does the aggregate supply.

5.2. Long Run Aggregate Supply (LRAS) is usually considered to be vertical. Hence, although changes in EM and EC may distort the equilibrium in the short run, the economy will eventually lean back to its original full employment level with a different price tag. We have appended a new section (Section: 6) in the article regarding this.

6. We move the step by step algorithm followed for Toda-Yamamoto approach of testing Granger Causality in the context of non-stationary time series to the appendix. We also reorganize 'Methodology' section with no sub-section.
7. We omit US from our analysis only because US deposit rate data are not publicly available. We collect annual deposit rate data of commercial banks from World Bank

data warehouse and US data are missing there. Moreover, US deposit rate data are not available in other popular public databases like Fed St. Louis economic data, IMF database or OECD statistics.

8. Reviewer’s understanding in this regard is similar to authors’ one.

Reviewer 2:

1. Reviewer has rightly pointed out that the borrowers are not the price setters unless it’s a monopoly. We apologize for the ambiguity we created here in the narratives. Our intended meaning in this regard is as follows: When the economy is growing at a rate g, we can assume that the producers of goods and services as a whole also get a g percentage point growth in their production, revenue and profit. If loans in the borrowers' account and output in real terms grow at the same pace (i.e., if $g = l$) then the accruals in loans can be served effectively by the enhancement in profit. If however $g < l$ then the borrowers have to manage extra money for interest servicing which can not be obtained from the growth in profit. As this is an economy-wide phenomenon not just for one single producer, the aggregate supply curve moves upward accordingly (as we recall interest expense as an inevitable cost of production [1], [2], [3]) and a higher equilibrium price level is set. Assuming a perfect competition, the borrowers/producers just have to follow the new equilibrium price thus set. In this regard, we have completely rewritten Section: 4 of the article.
2. Interest rate, inflation and exchange rate dynamics may be discussed under the following broad heads:
   A. Many factors add to interest rate.
   B. Interest rate adds to inflation.
   C. Inflation adds to exchange rate through international Fisher effect.

   Here, we confine ourselves to point B. Detailing point A and C simultaneously along with point B may go beyond the scope of a single study.

3. a) In the current study, structural breaks in the time series data are not taken into account. Incorporating structural break in our analysis at this point will irrevocably nullify all the testings that have been done, from unit roots to Block Exogeneity to ARDL. The study will then require new estimation techniques and may pose different results and interpretations which we think will go beyond the scope of a “Revise and Resubmit”.

   b) Now, we have reported the results of CUSUM test to judge the stability of the selected ARDL models which were not included in the previous submission. Results suggest that most of the ARDL models are stable.

   c) Now, we have reported the speed of adjustment and corresponding p-values for our selected ARDL models. Speed of adjustments are found to be negative in all the cases which implies some sort of mean reversion in the process and the corresponding p-values are found to be significant in almost all the reported cases of cointegration.

References:

[1] Barth, M. and V. Ramey, 2001, The Cost Channel of Monetary Transmission, NBER Macroeconomics Annual 2001, Cambridge, MIT Press, pp. 199-240.

[2] Dedola, L., Lippi, F., 2005, The monetary transmission mechanism: evidence from the industries of five OECD countries, European Economic Review 49, 1543-1569.

[3] Gaiotti, E., Secchi, A. (2006), Is there a cost channel of monetary policy transmission? An investigation into the pricing behaviour of 2,000 firms, Journal of Money, Credit and Banking, v. 38, 8, pp. 2013-2038.
